# A Treatise on Medico-Chirurgical and Topographical Anatomy, Considered Specially in Relation to Pathology, Medical Jurisprudence, Midwifery, and Operative Medicine

**Published:** 1845-07

**Authors:** 


### ART. XI.

*Traité d'Anatomie medico-chirurgicale et topographique, considérée spécialement dans ses applications à la Pathologie, à la Médecine légale, à l'Obstetricie et à la Médecine opératoire.* Par J. E. PETREQUIN, Chirurgien en Chef de l'Hôtel-Dieu de Lyon.—*Paris et Lyon,* 1844.

*A Treatise on Medico-Chirurgical and Topographical Anatomy, considered specially in relation to Pathology, Medical Jurisprudence, Midwifery, and Operative Medicine.* By J. E. PETREQUIN, First Surgeon to the Hôtel-Dieu, Lyons.—*Paris and Lyons,* 1844, 8vo, pp. 812.

IT is no disparagement of M. Petrequin's work to say that it almost entirely consists of facts long and familiarly known, for such must necessarily be the case with respect to any work on surgical and descriptive anatomy now published. This obvious fact of course dispenses us from the necessity of entering into a detailed analysis of the present volume, and enables us to limit our notice of it to mentioning the few points as to which M. Petrequin either is or claims to be original.

*Motions of the eyelids.* These motions give rise to a good deal of discussion. It is generally said that when the eyes are closed, the upper eyelid is depressed by its own weight. Malgaigne thought the explanation improbable, because during closure of the eyelids their external commissure approaches the nose to the extent of about one line, and because when, the eye being open, we look as perpendicularly downwards as possible, so as to see the under lip for example, the external commissure ascends higher than when the eye is shut, while the centre of the upper eyelid, on the contrary, is more depressed. M. Petrequin also denies that the upper eyelid is depressed by its own weight, because in paralysis of the orbicularis palpebrarum muscle, and after death, it descends but partially, and he thence argues that this motion must be effected by the orbicularis, seeing that paralysis of that muscle during life and the loss of its contractility consequent on death prevent its occurrence. Still greater difficulty attends the explanation of the motion of the lower eyelid. When the eye is open and looks straight forward, the lower eyelid is depressed and it is not supplied with a special muscle to produce that motion. Charles Bell considered that it was pushed downwards and outwards by the eyeball which was protruded by the contraction of the levator muscle; but to this Malgaigne objected, that when we look directly downwards the levator is not contracted, and yet the lower eyelid descends much more than in the preceding case, while if we strain the eyeball upwards the levator is very much contracted, but the lower eyelid ascends more than six lines above the lower brim of the orbit; M. Malgaigne avows that he is unable to explain those facts, but M. Petrequin says we can readily understand all the motions of the lower eyelid, when we recollect that it is connected by the membrane of Tenon to the globe of the eye, and must therefore follow all its motions. We must confess that we cannot see how this circumstance explains the "depression of the lower eyelid in the act of opening the eye," though it does enable us to understand how after restoration of the lower eyelid the artificial lid may enjoy a certain amount of motion as named in a case published by M. Jobert, (p. 97.) M. Petrequin in like manner refers the partial preservation of the motions of the eyelid in paralysis of the facial nerve to their connexion with the globe of the eye by means of the tunica albuginea (p. 160;) but this explanation is superfluous, for nothing is more familiarly known than that paralysis of the portio dura exists in very different degrees, and to a different extent in different cases, and that the orbicularis muscle, in common with other muscles of the face, for example those of the alæ nasi, are very frequently only partially affected.

*Adhesions of the eyelids to the globe of the eye, if of any extent,* are we believe, universally admitted to be irremediable; it is easy indeed to divide the adhesion with the knife, but a relapse has, so far we know, uniformly occurred during the progress of cicatrization. M. Petrequin has succeeded in remedying this acquired deformity in the following way. The principle of this method is to prevent the cicatrization of the opposed surfaces proceeding simultaneously, to have the healing process completed, or nearly so, on one surface before it has commenced on the other. In order to effect this object he pierces the adhesion at a suitable depth with a needle carrying a double ligature. The ligature corresponding to the eyelid is rather loosely tied, so that it shall very slowly divide the parts it includes, but the second ligature, which corresponds to the eyeball, is, on the contrary, very firmly constricted, and rapidly cuts through the adhesion. In this way the wound on the scelerotic may be healed before there is any exposed surface on the eyelid with which it can unite, and the denser and more extensive the adhesion, the easier it is, by regulating the tightness of the ligatures, to obtain an interval of several days between their separation. If the adhesion is very deep it must be divided by several operations, penetrating to a greater depth each time. The eyeball must be kept fixed during the process by means of carefully applied compression, as otherwise its motion and that of the eyelid might cause inflammation and premature separation of the ligature. (pp. 97-9.)

*Cleft palate.* The origin of cleft palate has been referred to very different causes, but no explanation has been hitherto offered of the remarkable fact that a fissure in the median line never occurs, except in the posterior part of the palate, and has never been observed anteriorly or in the lip. M. Petrequin accounts for this by the circumstance that the two inferior maxillary bones are not contiguous: as originally they are separated by the *inter-maxillary bone,* a fissure anterior to the palatine bones must therefore be situated between the inter-maxillary and one of the superior maxillary bones, and must be lateral at whichever side it may occur; but posteriorly the fissure must be median, as it exists between the two palatine bones which are not separated by any intervening bone. (p. 164.)

*Ranula.* M. Petrequin dissents from the prevalent opinion that ranula consists in a dilatation of the ducts of Wharton: 1st, because the symptoms of ranula are not similar to those of retention of saliva in the parotid gland; 2d, because the liquid it contains resembles, not saliva, but the glairy contents of some cysts; 3d, because the best mode of treating it is that which succeeds in cysts,—excision or obliteration of the cavity by irritant injections or by a seton; 4th, because, in ranula, incisions inevitably tend to close, while wounds of excretory canals, on the contrary, tend to become fistulous, and we never see after an operation on ranula the saliva flow abundantly during mastication, as occurs in salivary fistulæ; 5th, M. Brechet, on dissecting those tumours, always found that they were cysts occupying the submucous cellular tissue; and 6th, the progress of dilatation of Wharton's ducts by salivary calculi differs essentially from that of ranula: thus, in a case in which a large calculus was extracted from the duct, M. Petrequin observed that the incision, so far from closing, remained fistulous, and several months after the saliva could be abundantly expressed from t by pressure. (pp. 181-2.)

*Stammering.* We regret to find that M. Petrequin is still an advocate for attempting to cure stammering by a surgical operation, with which view he recommends and practises division of the attachment of the genio-glossi muscles to the lower jaw. As to the results of the operation M. Petrequin says, "it cures sometimes, often ameliorates, but frequently fails to produce any effect . . . We may generally hope to succeed when the stammering occurs with the labial, dental, or palatine letters, when the tongue has a tendency to be carried downwards or forwards between the teeth, when inspiration and expiration are free, and the patient is not too old; but relapses are greatly to be apprehended; the stammer may completely disappear after the operation, and subsequently return . . . To ensure a cure it is always useful to exercise the tongue in an appropriate way." (pp. 186-7.) This account is candid, but not very encouraging; and we apprehend that the special exercises of the tongue recommended after the operation would achieve every thing obtainable if practised without it.

*Mammary fascia.* In the region of the mammary gland, the *fascia superficialis,* according to M. Petrequin, presents a disposition which has hitherto been overlooked; it divides in fact into two layers which adhere firmly to the anterior and posterior surfaces of the gland respectively, and thus forms a fibrous envelope for the gland, a disposition which explains the pain accompanying swellings and inflammation of the breast. (p. 235.)

*Motion of the ribs.* It has been denied that the first rib enjoys any motion. M. Petrequin says the reverse can easily be proved by the following experiment. It is known that by carrying the shoulder forcibly backwards and downwards the subclavian artery may be compressed so as to arrest the circulation in the upper extremity. If when the artery is thus compressed between the first rib and the clavicle, the latter bone be elevated about four lines, the circulation is restored in the arm; but if the first rib is elevated and depressed during respiration, the circulation in the arm should alternately disappear and return as the individual inspires and expires, and in point of fact if a full inspiration is made, while matters are circumstanced as has been just described, the pulse in the brachial artery ceases at once, and consequently the first rib is really elevated during each inspiration. (p. 258.)

M. Petrequin's experiments on wounds and on suture of the intestines possess considerable interest, but as we shall shortly take a full review of this subject we shall not enter on it here.

*Glisson's capsule.* Few points in anatomy have given rise to more confusion and mistakes than has the description of the capsule of Glisson, and, as M. Petrequin attaches particular importance to his rectification of the errors of various writers respecting this structure, we shall give an abstract of his account of its structure, disposition, and uses. Underneath the serous covering of the liver lies its fibrous coat or *tunica propria.* At the transverse fissure of the liver this *tunica propria* sends off along the branches of the vena portæ, of the hepatic artery, and of the biliary canals, prolongations which form a kind of cylindrical sheath for each of these vessels and their several subdivisions ; it is those prolongations or sheaths alone that form the *capsule of Glisson,* and not the *tunica propria,* of which the capsule of Glisson is but a dependency. As to the use of the capsule of Glisson, M. Petrequin is of opinion that it is destined to facilitate the portal circulation. The liver is a compact organ not readily admitting of being distended, there is no heart to impel the blood through the vena portæ, nor are there even valves to sustain the column of blood in it ; its capacity is greater than that of the mesenteric artery to which it corresponds, and it is one of the vessels whose caliber is most variable, because from its relations to the digestive organs, it must admit of dilatation when fluids are absorbed, and be enabled to contract when those fluids have passed off. The various obstacles to the portal circulation here enumerated are counteracted, and the power of accomodating the capacity of the vessel to its contents is ensured, by its running in a fibrous sheath, and being quite independent of the tissue of the liver ; it can dilate or contract as the quantity of fluid traversing it increases or diminishes, and it can also contract so as to press on the blood it contains, and favour its circulation. (pp. 339-45.)

*Descent of the testicle.* M. Petrequin had an opportunity of dissecting a man aged 36, in whom one of the testicles had descended to the external ring, and the other remained within the abdomen. Unfortunately no description is given of the condition of the structure of the glands, it is not stated whether they were atrophied or had sustained any other alteration, nor are we told whether the virility of the individual was affected. We ourselves are convinced that detention of the testicles in the abdomen does not impair the procreative powers, but still as the dissections of those organs when so detained are few in number, we regret the omission in this instance. M. Petrequin is of opinion that, as late descent of the testicles is in the first place uncertain, and when it does occur, exposes the patient to hernia, or to the perhaps still greater inconvenience of having the gland arrested in the inguinal canal or at the external ring, it would be prudent when its absence from the scrotum is ascertained at birth to apply a truss in order to prevent the occurrence of those mischiefs. We refer our readers to our review of Mr. Curling's excellent work on 'Diseases of the Testicle' for a tolerably full consideration of this question. (pp. 374-7.)

Ossification of the septum of the corpora cavernosa occurs very rarely, and M. Velpeau is of opinion that it would occasion constant and distressing results, from which M. Malgaigne dissents, citing a case in which this affection caused no inconvenience in the ordinary condition of the organ, but produced great annoyance during erection. M. Petrequin saw a patient of M. Regnoli of Pisa, in whom ossification of the corpora cavernosa supervened on a contusion of the pelvis, and the organ was much incurvated when erect ; the ossified portion of the organ, which did not include the entire thickness of the corpora cavernosa, was excised, no bad symptom occurred, and the power of erection remained. (pp. 389-90.)

*Length of the urethra.* Authors differ extraordinarily in their statements respecting the *length of the urethra,* as appears from the following table which we borrow from M. Petrequin :

| | | | | |
|---|---|---|---|---|
| MM. Malgaigne . | . 5½ | inches to | 5¾. | (Anat. Chirurg.) |
| Velpeau . | . 5 | ,, | 7 | (Ib.) |
| Amussat . | . 7 | ,, | 8 | (Archiv. de Méd.) |
| Meckel . | . 8 | ,, | | |
| Whately-Ducamp-Begin | 7½ | ,, | 8½ | (Dict. de Méd. et Chirurg. Prat. t. xiv. p. 290.) |
| J. Cloquet . | . 7½ | ,, | 11 | (Anat Descript.) |
| Lisfranc . | . 9 | ,, | 10 | |
| H. Cloquet . | . 9 | ,, | 11 | (Anat. Descript.) |
| Sabatier . | . 10 | ,, | 12 | (Méd. Opérat.) |

In order to reconcile those discrepancies M. Petrequin examines the question under two heads : A, *the total length of the urethra,* and B, *the relative length of the urethra.* (pp. 399-400.)

A. *Total length.* This varies with the age, with individual peculiarities, and as the organ is erect or flaccid. But what is the mean length, and how is it to be determined? The method of injection is defective as its results vary with the force employed, and therefore merely indicate the dilatability of the urethra. The measurement with sounds has hitherto, according to M. Petrequin, been imperfectly applied, and with a view to greater accuracy, he has conducted the investigation both with straight and with curved instruments; with a straght instrument he generally found the length of the urethra between 5¾ and 6¼ inches, and with a curved instrument from 6¼ to 6¾ or even 7 inches; the difference arising from this, that the urethra not being naturally rectilinear, a straight instrument cannot be passed through it without effacing the angle between the bulbous and membranous portions of the canal; from these results M. Petrequin dissents from the statements of M. Velpeau, that when a catheter has been passed six inches into the urethra without elongating the penis it has arrived in the bladder, and that when we leave a gum-elastic catheter in that organ one inch of the instrument will be in its cavity if we introduce it to the depth of 6½ inches. (pp. 400-2.)

B. *Relative length.* By this term M. Petrequin means the estimation of the respective lengths of the several portions into which the urethra is ordinarily divided.

An exact determination of the average extent of the prostatic portion of the urethra is very important in relation to the operation of lithotomy. Boyer states it to be 15 or 16 lines; Littre 15; Ducamp and Blandin from 12 to 15; Senn 13; J. Cloquet 15. M. Petrequin agrees with Lisfranc, that the most exact approximate measurement is from 8 to 11 lines, but this length increases in enlargements of the prostate. The length of the membranous portion of the urethra is said by Boyer to be 12 lines, by Ducamp from 9 to 12, by Blandin 10, and by Lisfranc from 7 to 11. M. Petrequin has generally found the length of this portion of the urethra vary from 6 to 9 lines, when measured by its central axis; for it must be recollected that its parietes are of unequal length, its upper and longer surface measuring from 8 to 10 lines, while its inferior surface is about half that length, or 4 or 5, or sometimes 6 lines. The mean length of the prostatic and membranous portions of the urethra taken together is, according to Malgaigne, 13 lines, but varies from 11 to 15 lines; M. Petrequin has found it commonly vary from 14 to 18, and sometimes 20 lines, which agrees with the measurements of Mercier pretty closely. As to the bulbous and pendulous portions of the urethra, their *rectilinear*

measurement is 6 inches and 6 or 10 lines, and the *curvilinear* measurement 5 inches, or 5 inches and 4 lines. M. Petrequin agrees with Shaw and others, that the majority of strictures of the urethra occupy the posterior part of the bulb about $4\frac{1}{2}$ or 5 inches from the orifice of the urethra. (pp. 403-4.)

*Menstruation.* M. Petrequin's researches respecting the menstruation of females in the east of France have led him to adopt the conclusion, that one half menstruate between the ages of 13 and 15. The *critical age* occurs between 35 and 55, according to the following distribution.

| | | | | | | | |
|---|---|---|---|---|---|---|---|
| Between | 35 | and | 40 | years in | 1-8th of the whole number of women. | | |
| ,, | 40 | ,, | 45 | ,, | 1-4th | ,, | |
| ,, | 45 | ,, | 50 | ,, | 1-2d | ,, | |
| ,, | 50 | ,, | 55 | ,, | 1-8th | ,, | |

Thus fecundity ceases between 45 and 50, in about one half the entire number of women, and between 40 and 50 in about three fourths. As to the *duration of fecundity* he has found.

| | | | | | | | |
|---|---|---|---|---|---|---|---|
| The | minimum | from | 20 to 25 | years | in less than a quarter. | | |
| ,, | medium | ,, | 25 ,, 30 | ,, | more than a half. | | |
| ,, | maximum | ,, | 31 ,, 38 | ,, | about three quarters. (pp. 465-6.) | | |

*Prolapsus uteri.* Several cases are recorded in which polypus of the uterus has been mistaken for prolapsus of that organ. M. Pertrequin adds another to the list in the present volume. The patient, a woman aged 50, was considered not only by M. Petrequin, but by several other practitioners, to labour under prolapsus of the uterus, and as she was being exhausted by hemorrhage, the tumour which hung between the thighs, and measured 4 inches in one diameter, and 7 inches in another, was removed by ligature, and M. Petrequin was quite convinced that he had removed the womb; three weeks subsequently the woman died from pneumonia, and it was then ascertained that the tumour was a large polypus which had caused partial inversion of the uterus. (pp. 475-7.)

*Dislocation of the ulna.* Isolated dislocation of the ulna, that is the radius retaining its natural relations to the humerus, is considered by some authors impossible, and by all, we believe, very rare. M. Petrequin, on the contrary, thinks partial dislocation of the ulna a very frequent accident, and says he has ascertained that the radius usually remains in situ; he attaches considerable importance to this remark, as the mode of reduction should, he maintains, vary according as the radius is displaced or not. If both bones of the fore-arm are displaced, extension should be made while the forearm is pronated, so as to act on both bones in a right line; if the ulna alone is luxated, M. Petrequin thinks the reduction is best effected during supination of the forearm, and employs the radius as a lever and point d'appui, resting the epicondyle on his knee, and abducting the arm, which tends to separate the opposed surfaces of the humerus and of the ulna, and greatly facilitates the reduction. In this manner he reduced two dislocations, one of 7 weeks, the other of 101 days' standing. (pp. 589-90.)

*Necrosis.* J. Cloquet, Sanson, and others say that a bone is elongated during necrosis. M. Petrequin maintains the very reverse doctrine, as in two cases of necrosis of the radius he found that bone obviously shorter than the ulna, which projected considerably below it inferiorly, and thus inclined the hand to the radial side, contrary to the natural disposition of the parts. M. Petrequin ascribes the shortening in those cases to an arrest of development of the bone. We have no doubt that his observations are perfectly accurate, but we are equally certain that bones are much more frequently elongated during the progress of necrosis. The removal of a sequestrum from the radius is difficult, as the bone is almost entirely surrounded by tendons, nerves, arteries, and veins. M. Petrequin recommends the following mode of operating, which succeeded perfectly in two cases. By carrying an incision from the middle and posterior aspect of the radio-carpal articulation to the inferior third of the external border of the radius, we run along the dorsal branches of the radial vein, which are easily drawn aside; if the aponeurosis is now divided, the abductor and extensor of the thumb are exposed, and should be drawn outwards, separating them from the periosteum, so as to expose a sufficient surface of the bone to enable one or two small crowns of a trepan to be applied, in order to expose and extract the sequestrum. (pp. 605-6.)

*The groin.* M. Petrequin calls particular attention to the disposition of the *fold of the groin.* Its depth augments when the thigh is flexed and rotated inwards, and diminishes when the limb is extended; but it is never obliterated and is very deep and well marked in ascites. This permanence of the fold of the groin M. Petrequin attributes to the integuments being directly and firmly attached to the symphysis pubis by a radiate expansion of fibro-cellular tissue, which he names the *cutaneous ligament,* or *suspensory ligament of the fold of the groin.* The skin is also similarly but not so firmly attached to the spine and crest of the ilium. (pp. 660-1.)

*Inguinal and femoral rings.* The influence of the position of the trunk and of the lower extremity on the size of the inguinal and of the femoral rings, has not hitherto been accurately determined, according to M. Petrequin. From the result of numerous experiments on the dead body, he draws the following conclusions. Rotating the thigh outwards, and flexing it on the pelvis, considerably enlarges the external inguinal ring; simply flexing the thigh does not produce the same effect, but if in addition to flexing and rotating the thigh outwards the trunk is inclined forwards, the ring assumes its maximum size. Sitting on the hams, therefore, during the act of defecation, is dangerous in persons affected with constipation and predisposed to hernia, for the thighs being flexed and separated, and the trunk thrown forwards, the ring is at its maximum size. This posture should also be selected to favour reduction of a hernia when it is strangulated at the external ring, an event which M. Petrequin does not think of as rare occurrence as other authors do. (p. 668.) As regards the *internal crural ring,* M. Petrequin infers from experiments on the dead body, that it is most relaxed when the thigh is flexed, rotated inwards, and at the same time slightly abducted. Scarpa notices the relaxation of the ring when the thigh is flexed and rotated inwards, but he has not mentioned the additional influence of combining abduction with those motions. (p. 697.)

We have now given our readers a fair and we believe a full account of anything approaching to novelty contained in M. Petrequin's work. The book itself, however, is well worthy of perusal, and will be an useful work of reference for the practitioner. The descriptions are, we believe, universally accurate, though frequently not very clear, for the author has carried condensation to excess, a fault seldom to be found with French authors generally, whose endless prolixity contrasts strangely with the studied brevity of M. Petrequin. It would of course be a very unprofitable occupation of our space to give any specimens of the manner in which M. Petrequin arranges and describes familiar anatomical facts, but we may just say that we have hardly seen better or clearer descriptions than those he gives of the axilla, of the perineum, and of the anatomy of hernia.

---

